# Towards excimer-laser-based stereolithography: a rapid process to fabricate rigid biodegradable photopolymer scaffolds

**DOI:** 10.1098/rsif.2012.0300

**Published:** 2012-06-13

**Authors:** S. Beke, F. Anjum, H. Tsushima, L. Ceseracciu, E. Chieregatti, A. Diaspro, A. Athanassiou, F. Brandi

**Affiliations:** 1 Department of Nanophysics, Istituto Italiano di Tecnologia (IIT), via Morego 30, 16152 Genova, Italy; 2 Neuroscience and Brain Technologies, Istituto Italiano di Tecnologia (IIT), via Morego 30, 16152 Genova, Italy; 3 Department of Physics, University of Genoa, via Balbi 5, 16126 Genova, Italy; 4 Center for Biomolecular Nanotechnologies @UNILE, Istituto Italiano di Tecnologia, Via Barsanti, 73010 Arnesano (LE), Italy; 5 National Nanotechnology Laboratory (NNL), CNR—Istituto di Nanoscienze, via per Arnesano, 73100 Lecce, Italy

**Keywords:** laser photocuring, scaffolds, tissue engineering, biocompatibility, poly(propylene fumarate)

## Abstract

We demonstrate high-resolution photocross-linking of biodegradable poly(propylene fumarate) (PPF) and diethyl fumarate (DEF) using UV excimer laser photocuring at 308 nm. The curing depth can be tuned in a micrometre range by adjusting the total energy dose (total fluence). Young's moduli of the scaffolds are found to be a few gigapascal, high enough to support bone formation. The results presented here demonstrate that the proposed technique is an excellent tool for the fabrication of stiff and biocompatible structures on a micrometre scale with defined patterns of high resolution in all three spatial dimensions. Using UV laser photocuring at 308 nm will significantly improve the speed of rapid prototyping of biocompatible and biodegradable polymer scaffolds and enables its production in a few seconds, providing high lateral and horizontal resolution. This short timescale is indeed a tremendous asset that will enable a more efficient translation of technology to clinical applications. Preliminary cell tests proved that PPF : DEF scaffolds produced by excimer laser photocuring are biocompatible and, therefore, are promising candidates to be applied in tissue engineering and regenerative medicine.

## 1. Introduction

Owing to the immense research activity being devoted to tissue engineering and regenerative medicine [1–5], biomaterials used as scaffolds are expected to satisfy the emerging needs of rapidly reproducible platforms for increased clinical and research applications. Tissue engineering has been a rapidly expanding interdisciplinary field with the goal of growing tissues or organs directly on controlled microenvironments called scaffolds. Scaffolds are ubiquitously used as biomaterials alone or as a carrier for cells and nutrients. A biodegradable scaffold suggests that the implanted scaffold will gradually degrade *in vivo*, leaving behind the desired shape of the regenerated tissue without causing cytotoxicity [6]. Biocompatibility is the ability of a material to perform with an appropriate host response in a specific application [7]. Therefore, the degraded products of the material have to be non-cytotoxic as well. One of the major goals of tissue engineering is to provide rapid and accurate/reliable production of well-designed and functional scaffolds, since the day will come when biodegradable scaffolds will be mass-produced on a daily basis and routinely used as part of implants.

The advance of solid freeform fabrication techniques has significantly improved the ability to prepare structures with precise geometries using computer-aided designs and data from (medical) imaging [8,9]. These techniques include selective laser sintering, fused deposition modelling, three-dimensional printing and stereolithography (SL). In SL, the scaffold is produced by photocross-linking a polymer resin layer by layer. This may be a mixture of monomers or low molecular weight oligomers that can cross-link to form a long chain of solidified polymer upon irradiation. A large variety of photocurable and biodegradable materials [10–16], alone or as composites with ceramics, have been processed using SL. Poly(propylene fumarate) (PPF) is a promising candidate for tissue engineering of bones and cartilage. The biodegradability, cytocompatibility of the degradation products [17], high Young's modulus [18] and the unsaturation in the backbone of the polymer are some of the major characteristics of PPF, which lead to its diverse use in orthopaedic research.

SL, with a variety of modified operational procedures customized for specific applications, has been exploited by many authors [19–21], using different UV–visible wavelengths by pulsed or continuous wave laser beams as well as lamps, either by direct writing or by the image projection method.

The projection method is preferable in terms of production efficiency and simplicity of the apparatus because each layer can be fabricated with a single or few exposures to cover up to square centimetre area. Stencil masks, liquid crystal display or digital micro-mirror devices can be used in the projection method for variations in beam patterns [22–24]. There is, however, a very important issue of fabrication efficiency of biodegradable scaffolds that is crucial for the translation from scientific research to actual production. A fundamental factor determining the efficiency for scaffolds production with SL is the light power available on the resin to be photo-structured.

Excimer-laser-based projection photocuring [18] of liquid resins is a very promising method to increase production efficiency in SL. This is due to the high-power and well-shaped beams delivered by excimer lasers, which have no rivals in terms of industrial reliability among all other high-power UV-light sources, as demonstrated also by their intense use over the decades in the semiconductor industry. Moreover, excimer lasers can have wavelengths spanning all the deep-UV to UV range offering the flexibility necessary when using different materials for scaffold fabrication. The applied wavelength is of high importance as it determines the curing (penetration) depth of the laser pulses into the polymer and subsequently the time needed for scaffold production.

A prerequisite towards the development of excimer-laser-based SL is the investigation of high-resolution photocuring of suitable biocompatible and biodegradable resins. Recently, we demonstrated that deep-UV laser photocuring at a wavelength of 248 nm is an excellent technique to produce defined PPF : DEF patterns of submicrometre to micrometre resolution. The thickness of polymerized layers can be tuned in the micrometre range depending on the single pulse fluence, the number of pulses as well as the wavelength. The achievable aspect ratio was, however, limited by the penetration depth in the resin at the used wavelength [18].

In this paper, we report the development of a simple method to produce rigid biodegradable photopolymer scaffolds using excimer laser photocuring at 308 nm, that results in a curing depth tunable up to 100 micrometres which enables the production of structures with higher aspect ratios. The study is completed with mechanical characterization of the photocured polymer showing a Young's modulus comparable to most of biological hard tissues making such systems good candidates for scaffolds supporting bone formation. Bone cells can be divided into three cell types: osteoblasts, osteoclasts and osteocytes. Cell lines and primary cultures of these cell types have been widely used as models for studying processes such as bone formation, bone remodelling and skeletal development [25,26]. In order to test whether the scaffolds made by photocuring PPF : DEF with 308 nm light can indeed be tolerated by bone cells in culture, we chose a human osteosarcoma (HOS) cell line as our model system. Cell adhesion and viability are demonstrated, indicating that the produced scaffolds are suitable for assisting bone cell attachment and proliferation.

### 1.1. Experimental

All reagents and chemicals were purchased from Sigma-Aldrich and used as received, unless otherwise mentioned.

#### 1.1.1. Polymer synthesis

PPF was synthesized as reported elsewhere [27]. Briefly, fumaric acid was heated in excess of propylene glycol at 145°C with overhead mechanical stirrer. A Barrette trap was connected beneath the condenser, and water was collected as by-product. After 3–4 h of reaction, the temperature was increased to 180°C for 2 h to collect the unreacted propylene glycol and viscosity was checked. The polymer was purified by methylene chloride, followed by water and brine. Sodium sulphate was used as a drying agent of the organic phase, and finally, the PPF was made solvent-free via a rotary evaporator [28]. The outline of the synthesis process is shown in the electronic supplementary material, figure S1.

#### 1.1.2. Spectral characterization

FT-IR spectra were recorded with Bruker Vertex 70v in attenuated total reflectance mode. The characteristic peaks at 1717 cm^−1^ for ester linkage, 1647 cm^−1^ for vinyl moiety, 1458 and 1382 cm^−1^ for methyl stretching and at 1300 cm^−1^ for second alcohol are clear from the FT-IR spectrum of PPF (see electronic supplementary material, figure S2*a*). NMR spectra were acquired in deuterated chloroform on a Bruker 400 AV spectrophotometer. The chemical shifts at 6.89 ppm (vinyl protons), 5.34 (–CH), 4.28 (–CH2) and 1.37 (–CH3) confirms the structure of PPF (see electronic supplementary material, figure S2*b*).

#### 1.1.3. Molecular weight determination

The molecular weight characterization of PPF was performed by means of a multi-angle laser light scattering (MALS) detector online to a size exclusion chromatography (SEC) system using tetrahydrofuran (THF) as the mobile phase. This study was performed by using a multi-detector SEC–MALS system. The SEC system consisted of an Alliance chromatographic system from Waters (Milford, MA, USA) equipped with two online detectors: (i) MALS; and (ii) differential refractometer (DRI) used as concentration detector. The set-up of the multi-detector SEC system was serial in the following order: Alliance–MALS–DRI. In a multi-detector SEC system, the different online detectors have an intrinsic temporal delay because they are located in different positions of the fluidic path. Consequently, an alignment of the different signals is needed. The experimental procedure to determine the delay volume of different detectors has been described in the literature [29,30]. The weight average molecular weight was found to be 4600 Da.

The experimental conditions of the SEC–MALS chromatographic system in the molecular characterization of samples were the following: 150 μl of samples were injected at a flow rate of 0.8 ml min^−1^ at a concentration of about 40 mg ml^−1^ with degassed THF as mobile phase at 35°C.

#### 1.1.4. Sample preparation for photopolymerization

A photoinitiator, phenylbis (2,4,6-trimethylbenzoyl) phosphine oxide (BAPO), was dissolved in purified PPF and in the PPF : DEF (7 : 3 w/w) blend. The percentage of BAPO used was 1 per cent (w/w) in PPF and in PPF : DEF blend. A drop of the resin was spread on a glass slide and covered with a 130 μm-thick quartz slide in a sandwich manner. This way a few hundred micrometres-thick resin layer was formed between the glass and the quartz slides.

#### 1.1.5. UV transmission measurements

**Figure 1.**
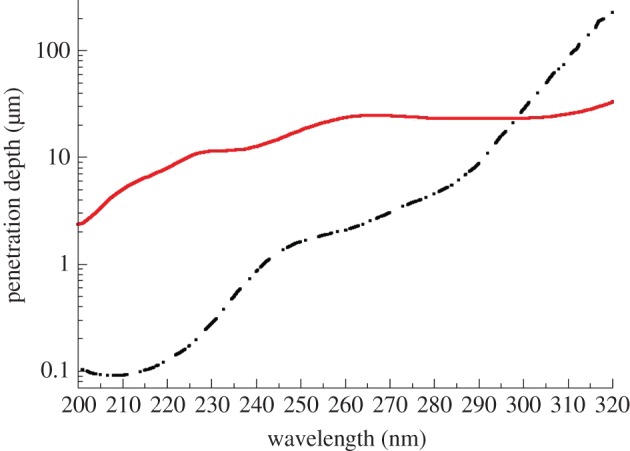
Deep-UV penetration depth of pure PPF (dashed-dotted line) and BAPO (solid line) photoinitiator at a concentration of 1% w/w. (Online version in colour.)

The deep-UV transmission spectra of PPF and of the BAPO photoinitiator before photoirradiation were recorded using a Varian (Cary 6000*i*) spectrophotometer, and with methanol as a solvent. The penetration depths in the deep-UV were calculated for the pure PPF and for the BAPO photoinitiator at 1 per cent w/w concentration in PPF, and are shown in figure 1. In our previous study, we used 248 nm where the penetration is 1.5 and 18.5 µm for PPF and BAPO 1 per cent w/w, respectively; so PPF dominated the laser light penetration. The penetration depth at 308 nm is 65 and 25 μm, for PPF and BAPO 1 per cent w/w, respectively; thus, BAPO is dominating the laser light penetration. The transmission spectrum of pure DEF is similar to that of pure PPF.

#### 1.1.6. Experimental set-up for photocuring

A schematic of the experimental apparatus used for high-resolution photocuring experiments is shown in the electronic supplementary material, figure S3. The light source is a XeCl excimer laser operating at 308 nm with a laser pulse duration of 20 ns and repetition rate of 1–100 Hz (CompexPro 110). We chose a repetition rate of 100 Hz throughout our experiments. The mask image is projected on the target using a demagnification of 4. The numerical aperture of the projection lens used is about 0.1. The actual pulse fluence is controlled by means of a variable attenuator. The proper positioning of the sample in the image plane is achieved by moving the sample in *x-*, *y-*, *z*-directions and using a CCD camera to monitor the process *in situ*. There was no movement in the *z*-direction during any of the experiments. After the irradiation, the quartz slides were washed in isopropanol to remove the non-polymerized resin, consequently the fabricated structures remained attached on the quartz plates for further investigations. The photopolymerized structures were subjected to characterization of curing depth, morphology and mechanical properties.

#### 1.1.7. Curing depth

Measurements were carried out on selected polymerized parts with a profilometer (Veeco Dektak 150) for the investigation of the curing depth.

#### 1.1.8. Mechanical tests

The mechanical properties of the photocured PPF and PPF : DEF blend were investigated by means of nanoindentation tests, performed on selected samples with a NanoTest 600 equipment (MicroMaterials, UK) at 24.0 ± 0.1°C on selected pillars with a pyramidal Berkovich tip. The maximum load was 2.0 mN; loading and unloading time were 2 and 1 s, respectively. The maximum load was kept for a long dwell time (30 s) in order to stabilize the viscous penetration and, this way, obtain a reliable unloading portion and elastic modulus. Sixteen measurements were conducted on each pillar, keeping a distance from the pillar rim of at least 20 μm, in order to avoid border effects on the measurements. The reduced elastic modulus *E*_r_ was calculated with the method of Oliver & Pharr [31]. The elastic modulus *E* was calculated by assuming a Poisson ratio *υ* = 0.3 for both PPF and PPF : DEF.

#### 1.1.9. Cell culture

Scaffolds were irradiated with UV for 30 min for sterilization, washed twice in phosphate-buffered saline (PBS) at pH 7.4, and coated with 0.1 mg ml^−1^ poly-d-lysine (MW 70 000–150 000 Da, Sigma) for 1 h at 37°C. Excess poly-d-lysine was removed by washing twice with PBS and once with Dulbecco's modified Eagle's medium (DMEM) with 8 per cent foetal bovine serum, 50 U ml^−1^ penicillin and 50 μg ml^−1^ streptomycin (Invitrogen). HOS cells were plated in DMEM at 2 × 10^5^ per 18 mm well containing one to three scaffolds, incubated in a humidified atmosphere with 5 per cent CO_2_ at 37°C and analysed after 2–10 days.

#### 1.1.10. Scanning electron microscope imaging

SEM images of the polymerized parts were taken using a JEOL JSM-6490 electron microscope. Both the as-fabricated scaffolds and cell-cultured scaffolds were analysed. A 20-nanometre-thick gold layer was sputtered over the polymerized arrays before the images were taken.

The cells grown on polymerized scaffolds were fixed in 1.2 per cent glutaldehyde (Sigma) in 0.1 M sodium cacodylate (Sigma) for 1 h at room temperature. After fixation, they were washed three times in 0.1 M sodium cacodylate for 10 min, and twice in water for 5 min. The cells were then incubated with increasing concentrations of ethanol (50%, 70%, 80%, 90% and 96%) for 10 min each. Subsequently, the cells were incubated in 100 per cent ethanol for 10 min three times, before they were moved to a glass Petri dish with 100 per cent hexamethyldisilazane (HMDS, Sigma) for 20 min. Finally, the sample was left overnight in 100 per cent HMDS until complete evaporation of the solution.

#### 1.1.11. Immunostaining and confocal microscopy

The cells grown on scaffolds were fixed in 4 per cent paraformaldehyde/4 per cent sucrose/PBS for 15 min at room temperature and washed three times with PBS. The cells were incubated with a primary antibody against α-tubulin (1 : 400, Invitrogen) in 5 per cent normal goat serum (Jackson Laboratories) 0.1 per cent Triton/PBS (0.1% PBT) for 1 h. Subsequently, the cells were washed four times in 20 mM phosphate buffer pH 7.4/0.5 M NaCl and were incubated with a fluorescent secondary anti-mouse antibody conjugated with Alexa488 (1 : 100, Invitrogen) and Phalloidin-TexasRed (1 : 400, Invitrogen) in 5 per cent NGS/0.1 per cent PBT for 1 h. The cells were washed three times in 20 mM phosphate buffer pH 7.4/0.5 M NaCl, once in PBS and once in water before they were mounted in ProLong Gold Antifade Reagent with DAPI (Invitrogen). Cell viability was tested using LIVE/DEAD Kits for Mammalian Cells (Invitrogen), according to manufacturer's protocol. Confocal images were obtained on a Leica TCS SP5 microscope using a 63× objective (Leica Microsystems).

## 2. Results and discussions

### 2.1. Curing depth-vertical resolution

For a systematic investigation of the curing depth as a function of the total irradiation dose (total fluence), a 1.2-mm round-shaped aperture was used as a mask. Owing to the 4× demagnification in the projection system, 0.3-mm-diameter areas were exposed to laser irradiation. Several arrays of photocross-linked structures were fabricated by applying fluence (*F*_p_) of 20 mJ cm^−2^ per pulse and varied the number of laser pulses (*N*_p_) while moving the sample in the transversal plane. This way, an array of pillars corresponding to a specific combination of laser pulse fluence and number of pulses resulting in a total fluence (*F*_t_ = *F*_p_ × *N*_p_) were produced and analysed. In all of our experiments, *F*_p_ = 20 mJ cm^−2^ was used for both the pillar and scaffold productions.

**Figure 2.**
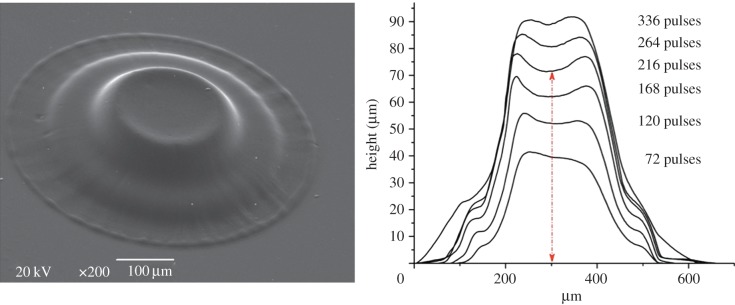
Pillar growth (PPF : DEF) as a function of number of pulses at 20 mJ cm^−2^. (Online version in colour.)

The height of the produced pillars and their profiles as a function of number of pulses is presented in figure 2. It is obvious that this technique provides a precise control on the height of the produced polymerized structures (curing depth). Note that because the surface profiles of pillars cannot be measured on the exact diagonal of the pillars, the presented diameters on the *x*-axis are just approximate values. The pillar heights were determined by measuring the distance between the quartz surface and the lowest part of the central ‘crater’ indicated by a red line in figure 2.

**Figure 3.**
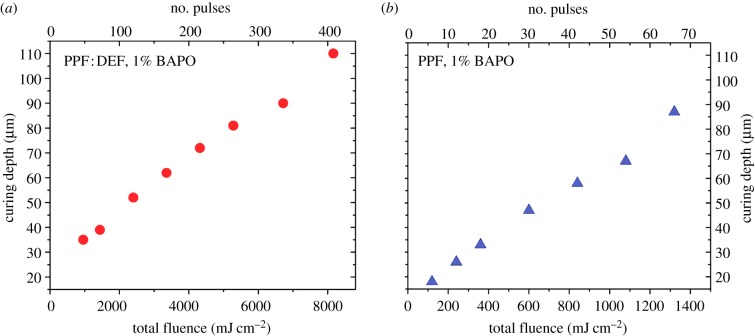
Plot of curing depth versus the total fluence: (*a*) PPF : DEF blend (7 : 3 w/w) with 1% BAPO and (*b*) pure PPF with 1% BAPO. (Online version in colour.)

Figure 3*a*,*b* presents the curing depth as a function of the total fluence for the PPF : DEF blend with 1 per cent BAPO and PPF with 1 per cent BAPO, respectively. From the plots of figure 3, it is found that for the PPF : DEF blend, the critical total fluence above which photopolymerization starts is 1000 mJ cm^−2^, which corresponds to a curing depth of 35 µm. For the PPF, the critical total fluence is fairly decreased to 120 mJ cm^−2^ with a penetration depth of 18 µm.

The data obtained from the profil measurements of the polymerized pillars show a correlation between the total fluence and the thickness of the cured layers, *C*_d_. In general, this correlation is theoretically expressed by the so-called working curve [10]

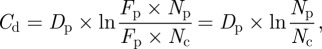

where *N*_c_ is the critical number of pulses at which photopolymerization starts for a given fluence per pulse (*F*_p_), and *D*_p_ is the penetration depth. This behaviour was indeed observed when 248 nm was used [18].

On the contrary, when 308 nm is used, the penetration depth of PPF is far higher than at 248 nm (figure 1); in addition, at 308 nm, the penetration depth of the photoinitiator (BAPO) is higher than that of PPF and PPF : DEF. The penetration depth at 308 nm is 65 and 25 μm, for PPF and BAPO 1 per cent w/w, respectively; therefore, when using 308 nm, BAPO dominates the laser light penetration. During the course of irradiation, the concentration of BAPO molecules continuously decreases as photons from the projected light break down the photoinitiator molecules into radicals along the penetration direction of the light. The radicals then bond the neighbouring polymers/monomers by breaking carbon double bonds. In this dynamic process, the correlation between the curing depth and total fluence is quasi-linear in contrast to the logarithmic in our previous study when 248 nm was used [18].

The higher total fluence necessary to achieve the curing of the PPF : DEF blend compared with pure PPF is attributed to the lower density of already-cross-linked material in the blend. Increasing the laser pulse fluence above *F*_p_ = 100 mJ cm^−2^ bubble formation in the polymer resin was observed during irradiation.

We would like to point out that figure 3 demonstrates the precise control of the curing depth of PPF at 308 nm with a few micrometres resolution being a fundamental prerequisite for the realization of an efficient SL apparatus. The broadening of the pillars on the substrate side is attributed to a spurious photocuring originating from: (i) large scattering at the quartz–resin interface and the large energy dose used to fabricate large cross-section pillars (this was produced in order to perform the nanoindentation measurements and allow repetitive measurement on different points on the same pillar) and (ii) lack of oxygen at the resin–quartz interface that limits the termination of the photocross-linking process (oxygen is known to be a very efficient cross-linking quencher). The non-flat pillar top surface is ascribed to non-homogeneous irradiation deep into the resin owing to small diffraction effects, which are enhanced by the large irradiation area and therefore large energy dose. This effect is not present when fabricating freestanding structures, as shown in the following sections.

### 2.2. Two-dimensional structures (with round-shaped and squared-shaped pores/channels)

On the basis of characterization of simple pillar structures, two-dimensional structures with accurate dimensions can be produced by projecting the image of a two-dimensional mask. The masks used for these experiments are quartz plates with metallic coatings of desired patterns.

**Figure 4.**
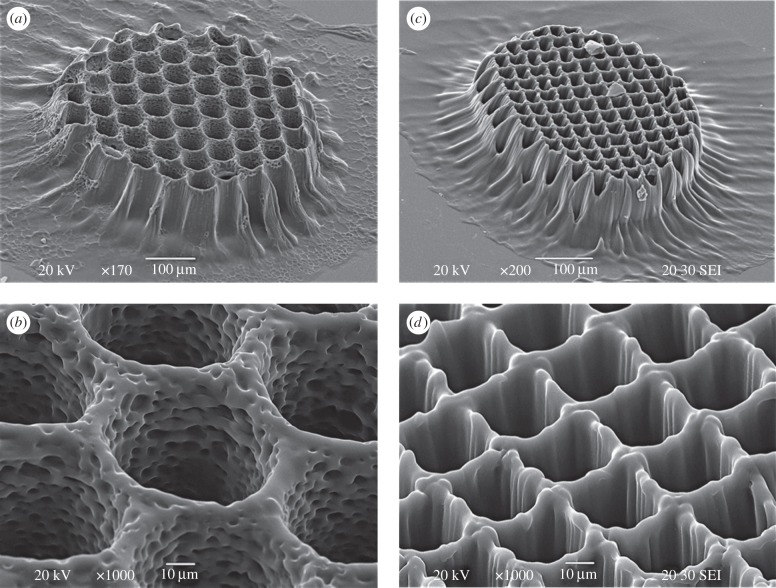
(*a*,*b*) SEM images of PPF : DEF polymerized grid with a round-shaped pore structures using *F*_p_ = 20 mJ cm^−2^ and 264 pulses. (*c*,*d*) SEM images of PPF : DEF polymerized grid with a squared-shaped pore structures applying *F*_p_ = 20 mJ cm^−2^ and 264 pulses.

Figure 4*a*,*b* shows a two-dimensional scaffold with round-shaped channels of photopolymerized grid (PPF : DEF with 1% BAPO) when applying 264 pulses.

The actual size of each round-shaped metallic spot on the mask is 220 µm and the spacing between them is 80 µm in all directions, which results in an exposure of 55 µm in diameter and 20 µm line spacing of the polymerized grid by using a demagnification of four. The estimated height of the grid based on figure 3 is about 80 µm, which is supported by SEM analysis (figure 4*a*). Figure 4*b* is a higher magnification SEM micrograph highlighting the internal porous structures (within the channels) that favours the cell adhesion for tissue engineering. By using masks with different dimensions, the produced channel/pore sizes can be easily tuned. A scaffold with squared-shaped pores is shown in figure 4*c*,*d*. The same experimental conditions were used (see electronic supplementary material, figure S3) except for the mask characteristics. The metal-coated squares of the mask have dimensions of 127 × 127 µm with a 27-µm spacing. After the demagnification of four, the exposure pattern is a squared grid with a line width of 7 µm and 27-µm spacing.

It is worth noticing the surface morphology of the produced scaffolds, which presents a porous or grooved micrometre pattern. These features are under further investigation, and may arise from to light channelling in the already cured material, that distributes energy dose with some micrometre scale pattern on the surface of the scaffolds.

### 2.3. Freestanding structures

**Figure 5.**
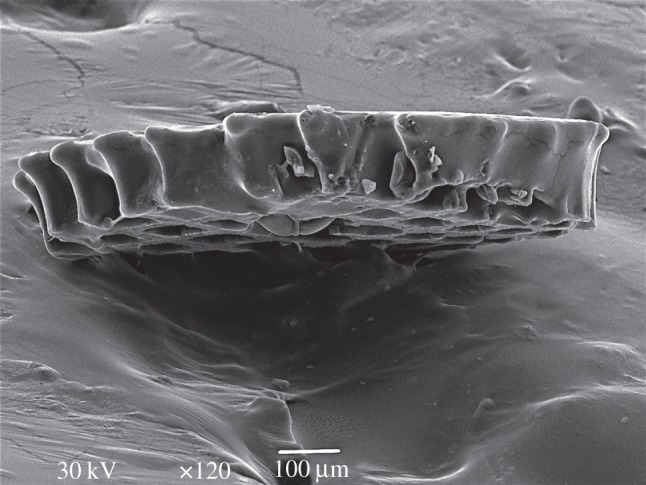
SEM images of PPF polymerized grid with a squared-shaped pore structures applying *F*_p_ = 20 mJ cm^−2^ and 350 pulses.

Producing freestanding polymer scaffolds are advantageous for several applications owing to their mobile and replaceable nature. In case of photocuring, this can be realized by directly irradiating the resin without the quartz slide on top. This means that the resin polymerizes and solidifies upon the laser irradiation without being adhered on a solid surface and, as such, stays ‘floating’ in the liquid resin. The SEM image shown in figure 5 proves that it is feasible to create two-dimensional scaffolds inside the liquid resin without a supporting surface. They can be formed directly in the liquid phase. Owing to the use of a higher viscosity resin along with a high repetition rate (100 Hz) laser irradiation, the accurate and precise solid phase scaffold formation can be achieved without a cumbersome liquid flow (which may occur between laser pulses in case the repetition rate and the resin viscosity are both low). When using a repetition rate of 100 Hz, the floating two-dimensional structure can be formed in the resin in a short-time (350 pulses in 3.5 s). Owing to its higher viscosity, PPF resin with 1 per cent BAPO was applied. After the irradiation, the floating structure was carefully removed and washed in isopropanol. Figure 5 shows an SEM micrograph where the scaffold is perpendicularly placed on a sticky tape and channels (pores) are visible on the bottom surface.

### 2.4. 2.5-dimensional structures

**Figure 6.**
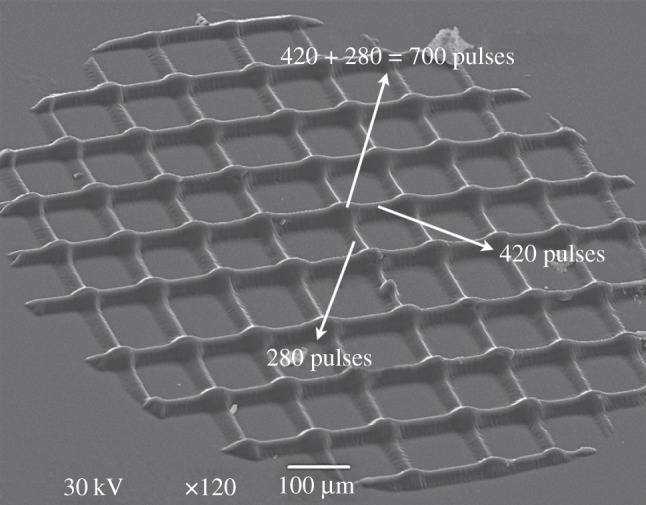
A SEM image of a 2.5-dimensional PPF : DEF polymerized grid when applying *F*_p_ = 20 mJ cm^−2^ and two different number of pulses in the vertical and horizontal directions resulting in the sum of them at the intersections.

To produce 2.5-dimensional structures, first a stencil mask was fabricated in-house using an excimer laser operating at 248 nm coupled with Optec MicroMaster micromachining workstation. Specifically, parallel slits were drilled in a thin metal foil. It is noteworthy that by using this technique any stencil mask with arbitrary continuous pattern can be fabricated. In the present case, the photocuring process comprises two consecutive exposures with a 90° rotation of the mask between the two exposures. This way, lines with different heights (curing depth) can be achieved. Consequently, at the intersections, the resin is exposed to a ‘double’ irradiation dose and thus the lattice becomes 2.5-dimensional. One example to produce a 2.5-dimensional lattice is presented in figure 6, where a burst of 280 pulses is applied in the first exposure and a burst of 420 pulses in the second one. As a result, at the intersections, the irradiation dose is equivalent to the sum of the two doses, i.e. 700 pulses. In summary, using these two different irradiation doses result in three different pulse dosages with the corresponding strut heights (figure 3).

### 2.5. Mechanical properties

**Figure 7.**
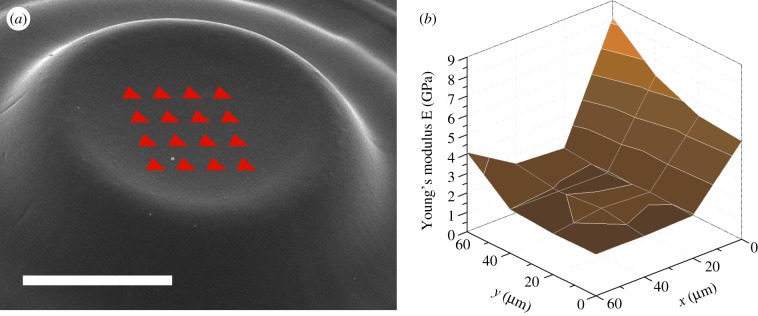
(*a*) A scheme of the indentation array on a typical pillar; the scale bar is 100 µm. (*b*) Example of measurements affected by the indentation location: the abnormally high value for *x* = 0 are due to curvature and border effect. (Online version in colour.)

Tests were conducted on the same pillars used for the profilometer measurements (figure 2). The pillars size allowed 4 × 4 arrays of indentations to be performed in each pillar. A scheme of the indentations array on a typical pillar is shown in figure 7*a*.

The boundary conditions for the correct analysis of indentations require the indented body to be flat and much larger than the indentation imprint. As can be seen in figure 7*a*, these are not fulfilled for all the indentations because of the curved surface in the border and the relatively short distance from the border itself.

As discussed previously, the curvature at the border is attributed to the inhomogeneity of the light penetration depth into the resin owing to diffraction effects. Therefore, the measured modulus can be affected by the indentation location, yielding unreliable values in the outward area. An example is shown in figure 7*b*. For this reason, data were filtered and abnormally high values corresponding to non-ideal testing conditions were excluded.

Overall, the average values measured (2.0 ± 0.5 and 2.8 ± 0.9 GPa for PPF and PPF : DEF, respectively) are in accordance with values in literature [32] and with those previous obtained with the 248-nm laser [18]. Results showed no clear dependence on the pillar height or laser parameters. This is consistent with the cross-linking mechanism upon the current set-up: the pillar height depends on the light coming from the pillar bottom creating enough cross-links to give solidity to the resin.

### 2.6. Morphology and structure of human osteosarcoma cells on scaffolds

**Figure 8.**
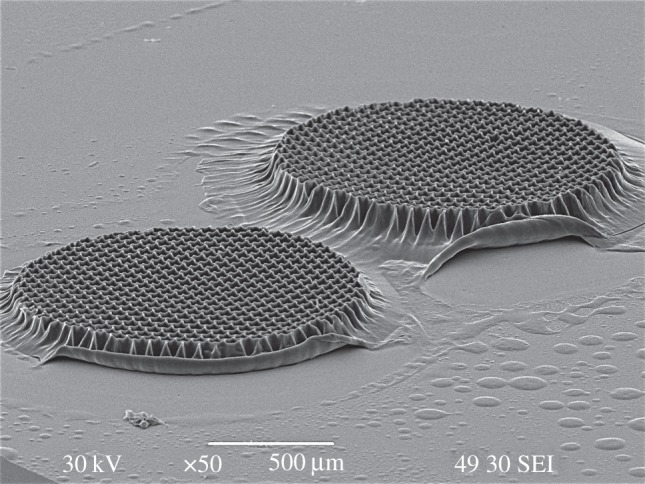
SEM images of larger (diameter of >1 mm) PPF : DEF polymerized scaffolds with round-shaped pore structures for cell tests using *F*_p_ = 20 mJ cm^−2^ and 264 pulses.

**Figure 9.**
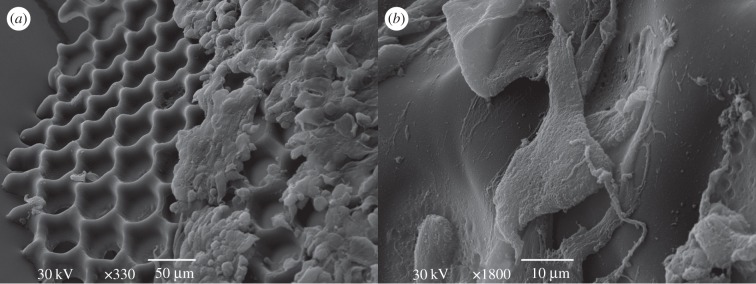
SEM images of HOS cells on polymerized scaffolds taken at (*a*) 330× and (*b*) 1800× magnifications.

For cell culturing, larger scaffolds (shown in figure 8) were used by changing the mask size in the experimental set-up (see electronic supplementary material, figure S3). The SEM images of the intact cells on the scaffolds taken after 48 h in culture (figure 9) show that the cultured cells on the PPF : DEF scaffold are undamaged, and adhere to the scaffold, indicating that the material is indeed cytocompatible. The adherent cells have morphology reminiscent of cultured fibroblasts [33]. HOS cells are spread over the surface of the scaffold (figure 9*a*,*b*) and grow in a similar manner to HOS cells cultured on a flat surface as the quartz surface surrounding the PPF : DEF scaffolds (electronic supplementary material, figure S4*a*,*d*).

**Figure 10.**
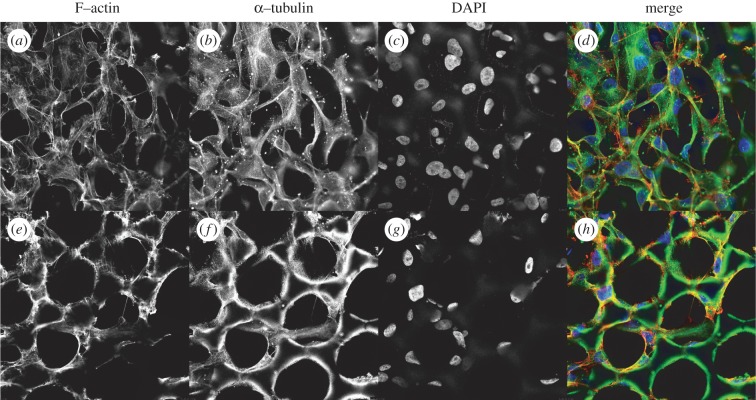
Confocal images of HOS cells on a polymerized scaffold showing immunostaining of F-actin (*a*,*e*, red in *d*,*h*), α-tubulin (*b*,*f*, green in *d*,*h*) and DAPI staining of nuclei (*c* and *g*, blue in *d*,*h*). (Online version in colour.)

HOS cells kept in culture on the scaffolds for 48 h were also examined by confocal microscopy for the intactness of the nuclei and for the expression of cytoskeletal proteins. Two major cytoskeletal proteins (actin and tubulin) were chosen for the immunofluorescence studies, as they outline the general morphology of the cells. Filamentous actin (F-actin), which is formed by polymerization of actin monomers, is enriched at the area adjacent to the cell surface membrane, often referred to as cortical actin network, and is present also in the cytoplasm. F-actin is required for various cellular processes such as membrane protrusion, stress fibre formation and attachments to the extracellular matrix through focal adhesions. The other cytoskeletal component analysed, the microtubules, consists of heterodimers of β- and α-tubulin. Microtubules play a crucial role in maintaining the cell structure, in the regulation of vesicle transport, in the formation of mitotic spindle and in cytokinesis during cell division. The expression and the localization of these structural proteins in the HOS cells growing on scaffolds were very similar to that seen in HOS cells that grew on the flat surface of quartz around the scaffold (see electronic supplementary material, figure S4*b*,*c* and S4*e*,*f*). Confocal images in figure 10 show F-actin (*a*,*e*), α-tubulin (*b*,*f*) and DAPI staining (*c*,*g*) of a scaffold in focus at two different *z*-axis, one at the top of the scaffolds (*a*–*d*) and one between the top and the bottom of the scaffolds (*e*–*h*). These images show that cells growing on the top surface of the scaffold (figure 10*a–d*) form an extended interconnected network, and adhere and grow also inside the pores of the scaffold (figure 10*e*–*h*). F-actin is organized in stress fibres and in a cortical actin network. Microtubules form bundles across the cells. DAPI staining of nuclei indicate the positions of the cells, some of them adhering to the inside walls or the edges of the wells in the scaffolds. Of note, the scaffolds themselves were autofluorescent at all three excitation/emission wavelengths used. Figure 10*d*,*h* shows the merged images of the three channels. HOS cells on the scaffold display a normal morphology, with intact nuclei and typical expression pattern of F-actin and α-tubulin.

Further confirmation of the viability of cells cultured on the PPF : DEF scaffolds, is achieved by the live/dead assay, monitoring apoptosis and the activity of specific enzymes. A movie of a three-dimensional projection image (see electronic supplementary material, figure S5), in which calcein (in green) is used to label live cells, indicates that the cells grow not only on the scaffold surface, but reach the bottom of the pores. Ethidium homodimer-1 (in red) is expected to mark dead cells, which were not present in all the samples analysed; it however stains the scaffold material unspecifically.

## 3. Conclusions and outlook

To our knowledge, this study is the first to present high-resolution photocross-linking of biodegradable PPF : DEF blend using excimer laser photocuring at 308 nm. The curing depth can be tuned up to 100 µm micrometres by adjusting the total energy dose (total fluence). Young's modulus of the photocured resin is found to be in the gigapascal range. Cell culture with HOS cells showed that PPF : DEF scaffolds produced by 308-nm laser photocuring are well-tolerated by the cells. The presented results demonstrate that the proposed technique is an efficient tool for the fabrication of stiff and biocompatible structures on a micrometre scale with defined patterns of high resolution in all three spatial dimensions. Therefore, these scaffolds are promising candidates for tissue engineering and regenerative medicine. These rapidly reproducible platforms can be of high importance for increased clinical and research applications.

The presented scaffolds are high aspect ratio two-dimensional and 2.5-dimensional structures, i.e. having three-dimensional geometries without suspended structures. Using the layer-by-layer SL method, the production of three-dimensional scaffolds with interconnected pores is feasible, with many potential clinical applications. The rapid photocuring time of a few seconds using 308-nm light for a single layer up to 100 micrometres or more, translates to minutes for centimetre-thick scaffolds. As a comparison, when using 248 nm light at the same laser pulse fluence, more than 30 times longer irradiation time is necessary [18]. For the lateral dimensions, the exposed area can readily be scaled up to square centimetres when large masks are used in image projection owing to the high power available from excimer lasers. We foresee that this approach will enable the translation of SL from the rapid prototyping arena to the actual production of three-dimensional biodegradable scaffolds, and enable an efficient translation of this technology to medical applications.
